# Nanosheet wrapping-assisted coverslip-free imaging for looking deeper into a tissue at high resolution

**DOI:** 10.1371/journal.pone.0227650

**Published:** 2020-01-10

**Authors:** Hong Zhang, Kenji Yarinome, Ryosuke Kawakami, Kohei Otomo, Tomomi Nemoto, Yosuke Okamura

**Affiliations:** 1 Department of Applied Chemistry, School of Engineering, Tokai University, Kanagawa, Japan; 2 Micro/Nano Technology Center, Tokai University, Kanagawa, Japan; 3 Course of Applied Science, Graduate School of Engineering, Tokai University, Kanagawa, Japan; 4 Research Institute for Electronic Science, Hokkaido University, Sapporo, Japan; 5 Graduate School of Information Science and Technology, Hokkaido University, Sapporo, Japan; 6 Department of Molecular Medicine for Pathogenesis, Ehime University Graduate School of Medicine, Ehime, Japan; 7 Exploratory Research Center on Life and Living Systems, National Institute of Natural Sciences, Aichi, Japan; 8 National Institute for Physiological Sciences, Aichi, Japan; 9 The Graduate University for Advanced Studies (SOKENDAI), Aichi, Japan; Nicolaus Copernicus University, POLAND

## Abstract

In order to achieve deep tissue imaging, a number of optical clearing agents have been developed. However, in a conventional microscopy setup, an objective lens can only be moved until it is in contact with a coverslip, which restricts the maximum focusing depth into a cleared tissue specimen. Until now, it is still a fact that the working distance of a high magnification objective lens with a high numerical aperture is always about 100 μm. In this study, a polymer thin film (also called as nanosheet) composed of fluoropolymer with a thickness of 130 nm, less than one-thousandth that of a 170 μm thick coverslip, is employed to replace the coverslip. Owing to its excellent characteristics, such as high optical transparency, mechanical robustness, chemical resistance, and water retention ability, nanosheet is uniquely capable of providing a coverslip-free imaging. By wrapping the tissue specimen with a nanosheet, an extra distance of 170 μm for the movement of objective lens is obtained. Results show an equivalently high resolution imaging can be obtained if a homogenous refractive index between immersion liquid and mounting media is adjusted. This method will facilitate a variety of imaging tasks with off-the-shelf high magnification objectives.

## Introduction

The ability to look deeper into a tissue is a constant demand from the biological or medical researchers who work with a fluorescence microscopy. Recently, a number of optical clearing agents that make tissues transparent have been developed, which ultimately allows tissue imaging to reach a deeper position [1–6]. In order to obtain exquisite structural information of tissues, such as neurons, a high magnification objective lens with a high numerical aperture (NA) is an essential component for imaging. However, the working distance (WD) of such a high resolving objective is always as short as about 100 μm, which determines the maximum focusing depth. For deep tissue imaging, while the tissue clearing technique provides a promising solution from the specimen aspect, it remains a practical challenge due to the restriction from the objectives aspect.

**Fig 1:**
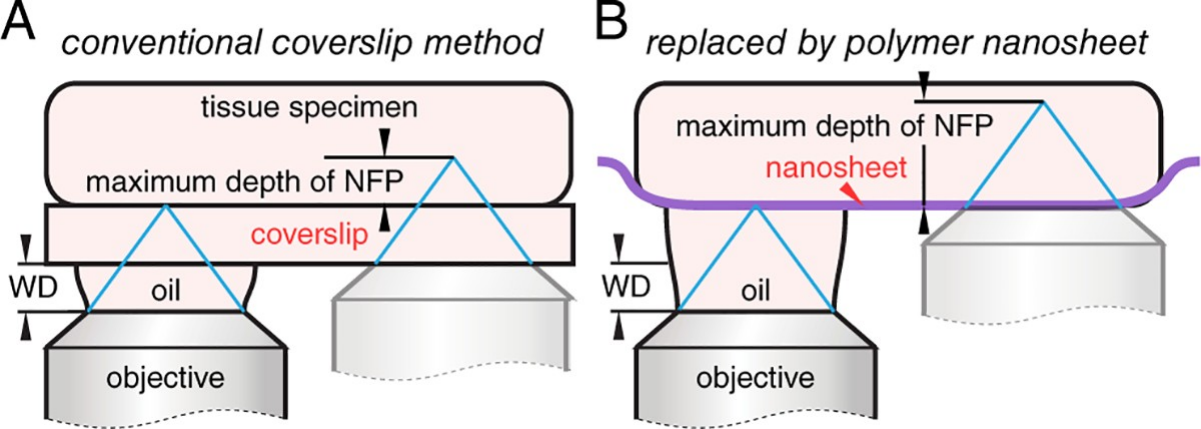
Schematics of coverslip method and nanosheet wrapping mount. (A) In a conventional coverslip method, an objective lens can only be moved until it is in contact with the coverslip. WD, working distance of an objective lens; NFP, nominal focal plane. (B) If the coverslip is replaced by a polymer nanosheet with a thickness of around 100 nm, an extra distance of 170 μm for the movement of an objective lens is obtained, namely a deeper maximum NFP can be expected.

Almost all the biological objectives are designed for a 170 μm thickness coverslip. For high magnification objectives (60× and 100×), liquid immersion lens, especially oil-immersion, is commonly used to obtain a higher resolving power, where the high refractive index (RI) of oil (RI: 1.515) compared to that of air (RI: 1.000) gives a higher NA of objectives. In this setup, an objective lens immersing in oil is located under the tissue behind a glass coverslip, where the lens can only be moved until it is in contact with the coverslip. The distance between the top lens of the objective and the surface of coverslip is known as WD; it roughly describes the maximum depth of the nominal focal plane that one can observe into a tissue specimen (Fig 1A). Here, we postulate that if the coverslip can be removed from the optical path, an extra distance for the movement of an objective lens would be obtained, and a deeper tissue imaging with a conventional setup and off-the-shelf objectives could be possible.

The absence of a coverslip when using an inverted microscopy seems impossible as it will cause the specimen falling onto the objective lens or at least a damaging exposure to the atmosphere or immersion liquids. In previous studies, we have developed a nanosheet wrapping method to address the issues of specimen shrinkage and movement in tissue and cell imaging [7, 8]. The term nanosheet refers to a transparent polymer thin film with a thickness of around 100 nm, less than one-thousandth that of a coverslip. Inspired by the use of plastic food wraps in our daily life, nanosheets have shown their capability to wrap various biological tissues. In this paper, we propose a nanosheet wrapping-assisted coverslip-free imaging method, where the tissue specimens are observed behind a polymer nanosheet, instead of a glass coverslip (Fig 1B).

## Materials and methods

### Fabrication of nanosheets

Silicon substrates (100) deposited with a 200 nm thermally grown silicon oxide layer (KST World Co., Japan) were cut to a size of 35×35 mm^2^ and treated with a piranha solution (1:3 (v/v) mixture of hydrogen peroxide and sulfuric acid). Poly(vinyl alcohol) (PVA, polymerization degree: ~500, 86.5%–89.0% hydrolyzed, Kanto Chemical Co., Inc. Japan) was dissolved in water at 10 mg mL^–1^. The solution was dropped on the substrate and spin-coated at 4000 rpm for 60 s (MS-A100, Mikasa Co., Ltd. Japan). Next, a solution of CYTOP (CTX-809SP, AGC Inc., Japan) dissolved in perfluorotributylamine at a concentration of 30 mg mL^–1^ was dropped on the PVA-coated substrate and spin-coated at the same conditions. The prepared substrate was immersed into water and the CYTOP nanosheet was released from the silicon substrate and floated on the surface of water. All the fabrication processes were conducted at room temperature (25°C) and a normal relative humidity (35% RH). The film thickness was determined by a scalpel scratch made on the re-supported nanosheets on silicon substrates, and measured using a stylus profilometer (DektakXT, Bruker Co., MA). The optical transmittance of a re-supported CYTOP nanosheet on quartz substrate was recorded with a UV-Vis spectrophotometer (UV-2600, Shimadzu Co., Japan).

### Mechanical tests on nanosheet wrapping

With the help of a homebuilt wire loop (diameter: ~28 mm), CYTOP nanosheets can be lifted out of water and supported in the air. The specimen was gently placed in the center of a coverslip with a diameter of 25 mm (0.13–0.16 mm thickness, AS ONE Co., Japan), and then wrapped with a nanosheet. To test the bearing capacity of a nanosheet, a round magnetic tape (diameter: 10 mm, thickness: 0.5 mm, KOKUYO Co., Ltd. Japan) was used as a model tissue and wrapped with a CYTOP nanosheet. A permanent magnet cylinder (diameter: 10 mm, thickness: 10 mm, remanence magnetic flux density: 480 mT) was approached from a distance of 10 mm with a speed of 2.0 mm min^–1^ until the nanosheet ruptured. This process was performed on a horizontal universal testing machine and the force was recorded by a digital force gauge (FSA-0.5HK2 and ZTA-2N, IMADA Co., Ltd. Japan). The photos of the nanosheet wrapping process, the nanosheet wrapped agarose gel and brain slice were taken using a digital camera G7 X Mark II (Canon Inc., Japan). The photos of the mechanical test were captured using a portable microscopy (3R-MSUSB401, 3R SOLUTION Co., Japan).

### Contact angle measurements on nanosheets

A CYTOP nanosheet was prepared on a silicon substrate using spin-coating as described above. Polydimethylsiloxane (PDMS) was chosen as a comparison polymer to prepare the nanosheet. Specifically, silicone elastomer Sylgard 184 (Dow Chemical Co., MI) with a mixing ratio between base and curing agent of 10:1 (w/w) was dissolved in hexane at 12 vol%. The solution was dropped on the silicon substrate and spin-coated at 4000 rpm for 60 s, and then cured at 80°C for 2 h. The static contact angle was obtained on a contact angle meter (DMe-201, Kyowa Interface Science Co., Ltd. Japan) at room temperature (25°C) and a normal relative humidity (35% RH). The testing liquids included water, mineral oil (SMR-100, ULVAC Inc., Japan), and immersion oil Type NF (RI: 1.515 at 23°C, Nikon Instruments Inc., Japan). To demonstrate the chemical resistance ability of a nanosheet, the contact angle was measured after being in contact with immersion oil for 1 h as well. It should be noted that the immersion oil Type NF is composed of 55–65% diphenyl ether, 25–35% polybutene, and 5–15% mineral oil, as given by the datasheet from Nikon Instruments Inc.

### Water retention effect of nanosheet wrapping

Blue dextran (*M*_w_: 2000 kDa, Sigma-Aldrich Co. LLC, MO) containing sodium alginate (Kanto Chemical Co., Inc.) aqueous solution at 20 mg mL^–1^ was poured into calcium chloride solution at 2 wt% with a ratio of 1:2 (v/v). An alginate hydrogel was formed after stirring overnight. Hydrogels to be wrapped were punched into a cylindrical shape with a diameter of 10 mm and thickness of ~5 mm. The original weight and weight of wrapped samples at 1 h time intervals over 24 h were measured as *W*_0_ and *W*_t_, respectively. Then, the sample was completely dried in an 80°C oven and weight as *W*_d_. The water retention ratio was thus obtained by (*W*_t_−*W*_d_)/(*W*_0_−*W*_d_)×100%. The test was conducted in a constant temperature and humidity room at 24°C and 40% RH.

### Imaging on model tissues

Fluorescent beads with a diameter of 2 μm (Fluoresbrite YG, Polysciences, Inc., PA) were used to measure the accessible focal position under different imaging conditions. A 0.5 wt% agarose gel (Agarose-I, Dojindo Laboratories Co., Ltd. Japan) embedded with 1×10^7^ particles mL^–1^ beads (diameter: 5 mm, thickness: ~2 mm) was wrapped with a nanosheet, and placed on a perforated bottom dish (35 mm diameter) with the nanosheet side downwards. A 1 μm thickness polystyrene film loaded with Nile red was attached in between the gel and the nanosheet as a height reference (*z* = 0). Image stacking was acquired with a 1 μm *z*-step from the surface of nanosheet to a depth of 400 μm over an area of 212×212 μm^2^. As a control group, a coverslip supported sample was observed as well.

### Spatial resolution with nanosheet wrapping

In order to evaluate the spatial resolution, the point spread function (PSF) was measured. Green fluorescent nanoparticles (NPs) with a diameter of 200 nm (Fluoresbrite YG) were embedded in agarose gel with 1×10^9^ particles mL^–1^. Images of fluorescent nanoparticles within the volume of a 42×42×5 μm^3^ cube located at the center of imaging field were obtained at every 50 μm. Ten particles at each depth were arbitrarily chosen, and the full width at half maximum intensity (FWHM) values were measured by fitting their fluorescence intensity profiles along the *x*-axis and *z*-axis around the central intensity using a Gaussian function. This analysis was repeated three times and the averaged value was adopted. In most cases, gels were treated to be transparent by LUCID (RI: 1.496, Nikon Instruments Inc.) for 6 h or further modified with RapiClear (RI: 1.515, Sunjin Lab, Taiwan) overnight at 37°C. The RI of gels was measured with a pocket refractometer (PAL-RI, ATAGO Co., Ltd. Japan).

### Tissue imaging on brain slices

Thy1-EYFP-H transgenic mice (8–12 weeks old, female, *n* = 4) were used in this study. The expression of EYFP in the H-line mouse enabled us to observe green fluorescent neurons in brain slices. This study was carried out in accordance with the recommendations in the Guidelines for the Care and Use of Laboratory Animals of the Animal Research Committee of Hokkaido University and all protocols were approved by the Institutional Animal Care and Use Committee of National University Corporation Hokkaido University (Permit Number: 17–0077). All experiments were performed under general anesthesia, and all efforts were made to minimize suffering. Mice were anesthetized with pentobarbital sodium, and transcardially perfused with phosphate-buffered saline (PBS) followed by 4% formaldehyde in PBS. The fixed brains were removed and sliced using a Brain Matrices (Muromachi Kikai Co., Ltd. Japan) into 1 mm sections. These slices were cleared with LUCID or further modified with RapiClear, and wrapped with a CYTOP nanosheet, using the same method as described above. Polystyrene films loaded with Nile red were used as a height reference. Image stacking was acquired with a 1 μm *z*-step from the surface of nanosheet to a depth of 400 μm over an area of 212×212 μm^2^. Five slices from different mice for each optics condition were examined to ensure the result reproducibility.

### Microscopy and image acquisition

All the observations were taken on an inverted confocal microscopy with a LU-N4 laser unit (A1R+, Nikon Instruments Inc.), and the pinhole was fixed to be 0.8 AU (physical diameter: 24.3 μm, the pinhole calculated from the longest wavelength of emission here). A 60× oil-immersion objective (CFI Apo TIRF 60×H, NA: 1.49, WD: 0.12 mm, with correction collar at 0.17 mm with 23°C) and a 60× water-immersion objective (CFI Plan Apo VC60×WI, NA: 1.20, WD: 0.27 mm, without correction collar) were used. Laser lines at 488 and 561 nm were used for Fluoresbrite YG as well as EYFP and Nile red, respectively. Correspondingly, fluorescence signals in the wavelength range of 500–550, and 570–620 nm were detected via a photomultiplier tube. Immersion oil Type NF was used. In some cases, the immersion oil was replaced by TDE aqueous solution with a RI of 1.496. TDE (Sigma-Aldrich Co. LLC) was dissolved in water at various volume concentrations, and the standard RI curve was measured using an Abbe refractometer with a filter of 589 nm at 23°C (DR-M2, ATAGO Co., Ltd.). For facilitating the comparison of different optics with nanosheet wrapping, all the images were acquired with the same laser power, detector gain value, and scanning speed through NIS-Elements C software (ver. 4.51, Nikon Instruments Inc.). For 3D deconvolution, the acquisition data was computationally processed by Huygens Essential software (ver. 18.04, SVI BV, Netherlands) using a routine maximum likelihood estimation method. A measured PSF in the same optics condition acquired from 200 nm NPs as mentioned above was used for the input of deconvolution calculation.

## Results and discussion

**Fig 2:**
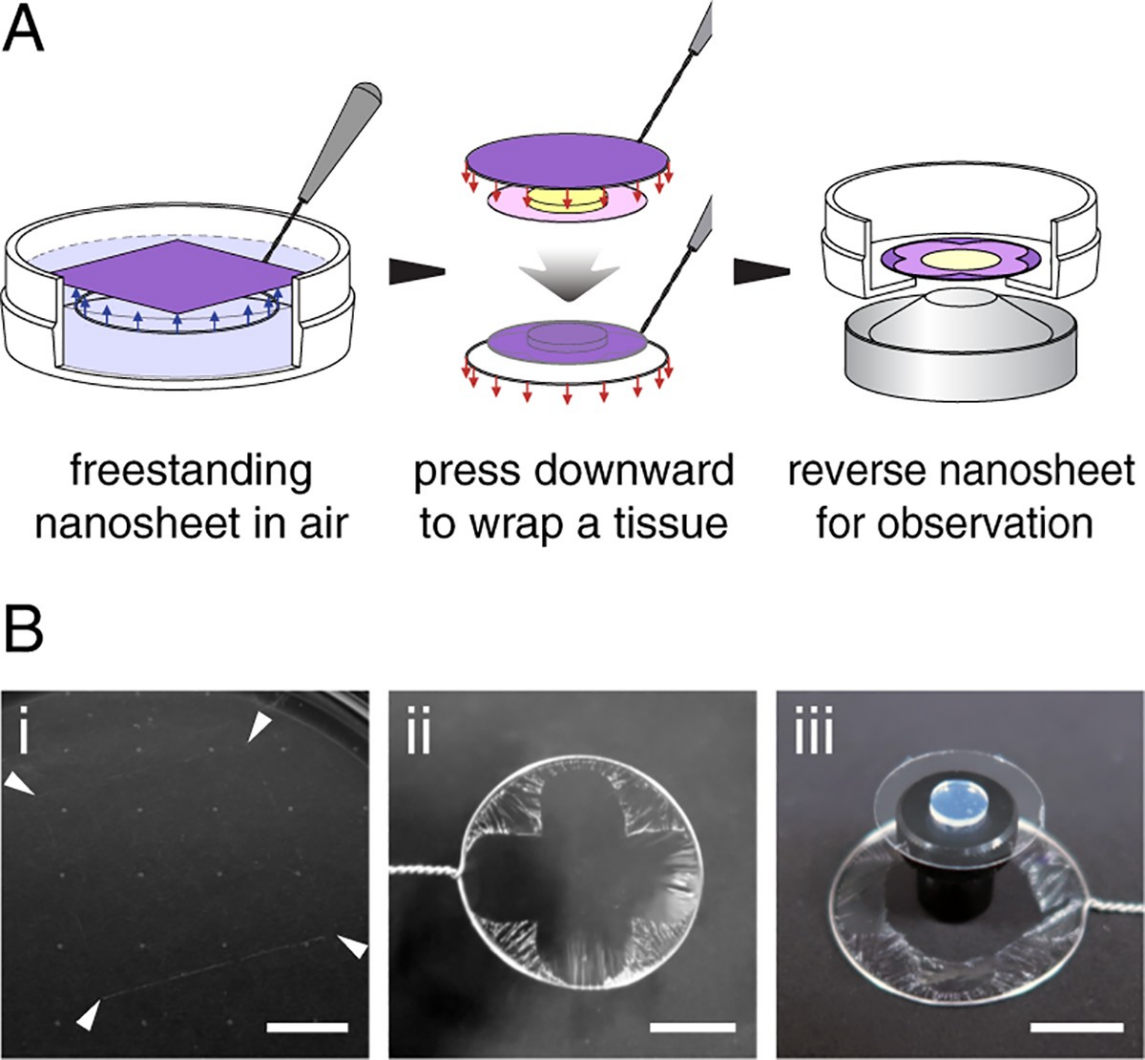
Nanosheet wrapping process. (A) Schematic of nanosheet wrapping process. A tissue specimen is placed on a coverslip in advance. The nanosheet is transferred from the surface of water to wrap the coverslip with the help of a wire loop, which is then reversed and bonded to a perforated bottom dish for observation. (B) Photos of a typical wrapping process. From (i–iii): a CYTOP nanosheet floated on the surface of water (arrows indicate the corner of nanosheet); the same nanosheet supported by a wire loop in the air; and an agarose gel (~2 mm thickness) wrapped with a nanosheet. Scale bar, 1 cm.

Perfluoro(1-butenyl vinyl ether) (CYTOP), an amorphous fluoropolymer with good coatability, is chosen as the material for constituting nanosheets. Similar to Teflon, CYTOP offers superior hydrophobicity and oleophobicity, which is resistant to almost all chemicals. Using a spin-coating method, the thickness of CYTOP nanosheet is adjustable and is set to be 130 nm in this study (S1 Fig). A water-soluble polymer layer is coated before CYTOP casting, and nanosheet can then float on the surface of water with dissolution of the underlying layer [9–14]. Nanosheet wrapping on a tissue specimen is accomplished with the help of a wire loop, while the marginal part of the nanosheet can automatically adhere to any surface (Fig 2). The protocol for nanosheet fabrication and wrapping is also provided in Supporting Information (S1 Text). A nanosheet wrapped mouse brain slice illustrates the high transparency of a nanosheet (S2A Fig). We demonstrate that a 130 nm CYTOP nanosheet has almost 100% transmittance over the wavelength range from 300 to 800 nm, which determines its optical reliability for biological imaging (S2B Fig).

**Fig 3:**
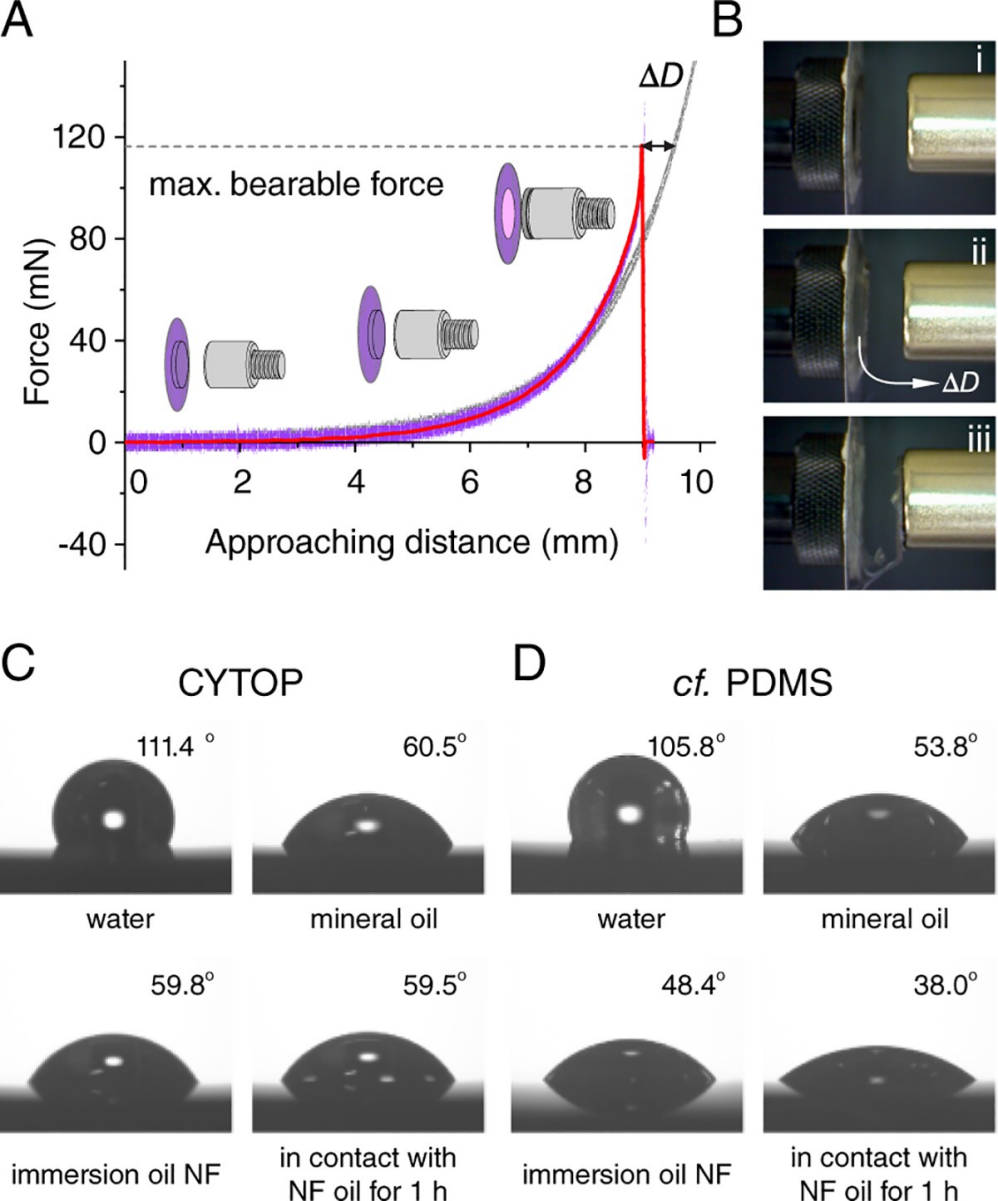
Mechanical robustness and chemical resistance of CYTOP nanosheet. (A) The recorded force during the process of a magnet approaching, where the applied force is constantly increased until the nanosheet breaks (purple line, and smoothing is shown in red). Compared to the control group without wrapping (gray line), the stretchable distance of a nanosheet, ΔD, is about 0.6 mm. (B) Snapshots taken in sequence (i–iii) during the test process, which are 40 mN is applied under the elastic region of nanosheet, 116 mN is applied to achieve a maximum bearable force of nanosheet, and the moment that nanosheet is broken with a further magnet approaching. (C) Contact angles with a variety of liquids on a silicon substrate supported CYTOP nanosheet (thickness of 130 nm), including water, mineral oil, immersion oil Type NF, and the contact angle measured after in contact with NF immersion oil for 1 h. (D) The same contact angle measurement is conducted on a silicon substrate supported PDMS nanosheet (thickness of 130 nm) as a comparison.

One may wonder whether a CYTOP nanosheet can support the weight of a tissue specimen when it is mounted with the nanosheet side downwards. CYTOP is a mechanically stretchable, and durable (Young’s modulus: 1.3 GPa) fluoropolymer [15]. In fact, the CYTOP nanosheet shows a high degree of robustness in normal handling. With a non-contact measurement, we find the maximum force that a 130 nm CYTOP nanosheet can sustain without failure is about 116 mN (Fig 3A and 3B). No plastic deformation is observed if the applied force is less than 40 mN, which means that wrapping a tissue specimen with a weight of 4 g or less is highly assured to cause no difficulties. The relative brain weight for an adult mouse (more than 8 weeks old) is about 10% of its body mass, and the whole brain weight is no more than 2 g [16]. Thus, we know that a nanosheet is sufficiently strong to replace a coverslip in a common tissue imaging.

Moreover, CYTOP is highly resistant and inert to almost all chemicals. The composition of a commercial synthetic immersion liquid oil is complex and different among various microscope manufactures, which generally contains mineral oil, polychlorinated biphenyl, phenyl benzene, or diphenyl ether [17]. Polydimethylsiloxane (PDMS) is chosen as a comparison polymer as its well-known chemical resistance ability, and the contact angle measurement is conducted on silicon substrate supported CYTOP and PDMS nanosheets (both thickness of 130 nm). After in contact with immersion oil for 1 h, the contact angle with immersion oil of PDMS nanosheet significantly decreases from 48.4° to 38.0°, indicating the immersion oil infiltrates into the PDMS nanosheet with time. This result agrees with the previous study that a PDMS device swells with the immersion oil and turns into non-uniform shape [18]. For CYTOP nanosheet, however, the contact angle keeps constant at 59.6°±0.2°, and no contact angle change is observed, which exhibits an excellent chemical resistance when CYTOP nanosheet is exposed to contact with immersion oil for a long time (Fig 3C and 3D).

**Fig 4:**
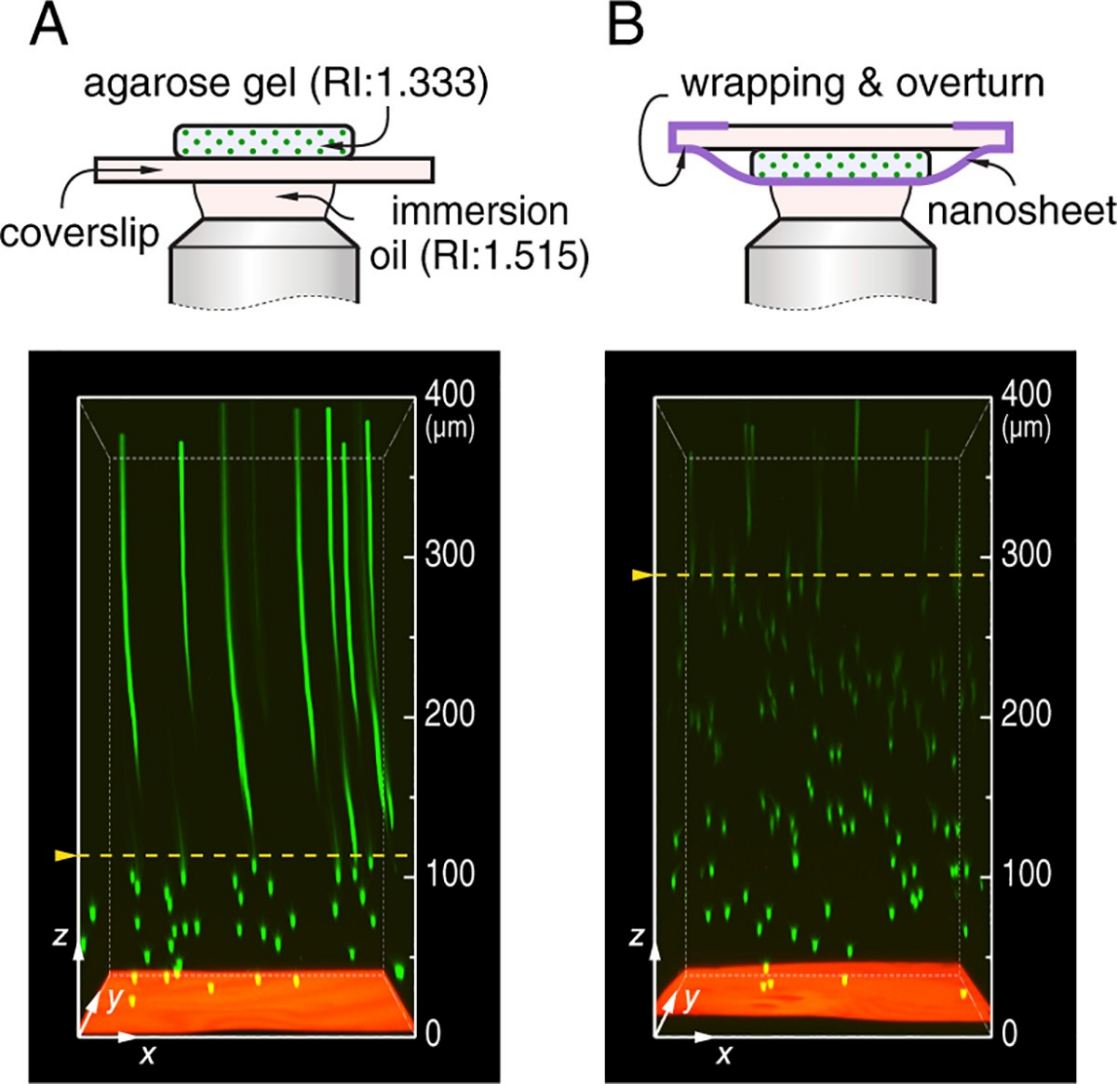
Coverslip-free imaging on a model tissue. (A) With a conventional setup, a coverslip supported agarose gel embedded with 2 μm green fluorescent beads is observed from the upper surface of coverslip to a depth of 400 μm, and a 1 μm thick polystyrene film loaded with Nile red is applied as a height reference at *z* = 0. (B) Imaging on a pristine agarose gel with nanosheet wrapping mount. The corresponding schematics of optics conditions are shown above, and yellow dashed lines indicate the maximum depth of focal plane in each condition. Imaging area in *xy* plane is 212×212 μm^2^, and depth is labelled at the side.

We use a high magnification oil-immersion objective lens (60×) with WD of 120 μm and NA of 1.49, if not otherwise stated. An agarose gel embedded with green fluorescent beads with a diameter of 2 μm is used as a model tissue. With a coverslip, the focal plane in agarose gel is limited to 115 μm, very close to the WD of the objective lens. The elongated beads at deeper regions are artifacts arising from the collision between objective and coverslip (Fig 4A). In the case of nanosheet wrapping, however, the accessible depth of identifiable beads is much increased, which verifies the principle of our method well. In this case, the refractive indexes of agarose gel and immersion oil are 1.333 and 1.515, respectively, and thus such a RI mismatch causes a severe spherical aberration and the brightness of beads deteriorates rapidly (Fig 4B).

**Fig 5:**
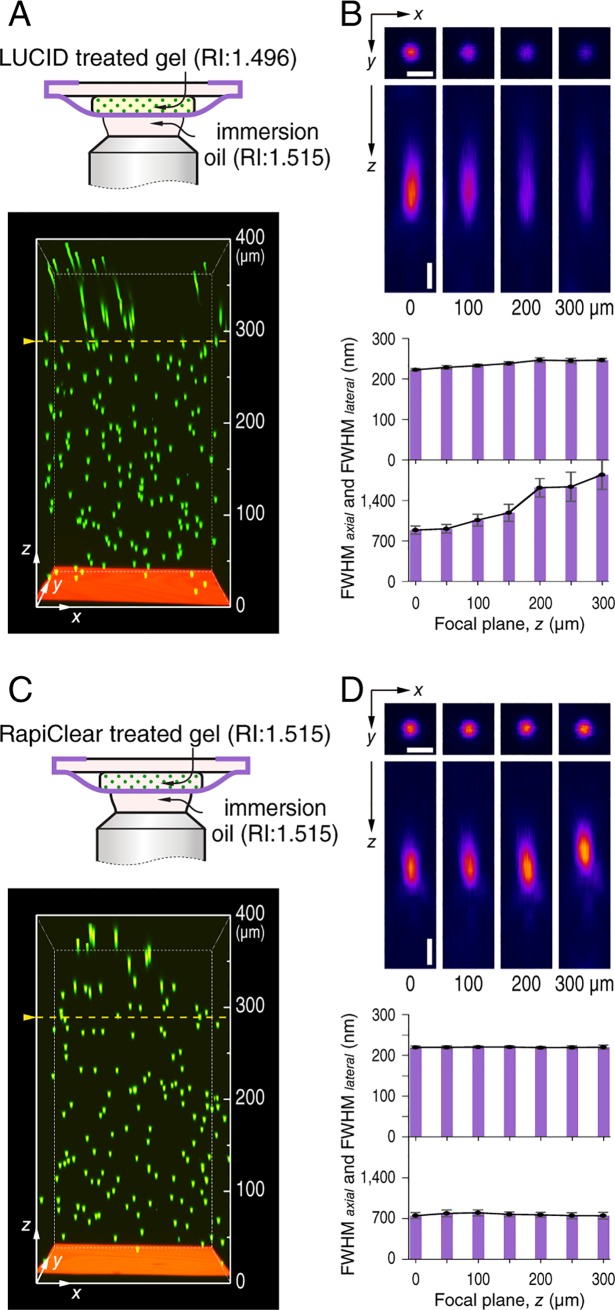
Coverslip-free imaging on a model tissue with adjusted optics conditions. (A, C) With nanosheet wrapping mount, imaging tests with an agarose gel treated with LUCID, and further modified with RapiClear are conducted. The corresponding schematics of optics conditions are shown above, and yellow dashed lines indicate the maximum depth of focal plane in each condition. Imaging area in *xy* plane is 212×212 μm^2^, and depth is labelled at the side. (B, D) Spatial resolutions at different depths of nanosheet wrapping mount. 200 nm NPs are embedded in agarose gel and images are obtained at every 50 μm. Scale bar, 500 nm; and depth is labelled below. Representative NPs at each depth are arbitrarily chosen (*n* = 3 independent tests; and 10 NPs are analyzed for each test), and FWHMs along the intensity profile in *x*-axis and *z*-axis are measured as shown in histograms (mean ± SEM).

After treated using a clearing agent, ilLUmination of Cleared organs to IDentify target molecules method (LUCID) [19, 20], the RI of agarose gel is adjusted to 1.496, and the depth of beads that can be well identified extends to almost 300 μm, an increase of a factor of 2.5 as compared to the coverslip case (Fig 5A). In an ideal optical path, the extra WD of 170 μm would completely convert to focusing depth. Here, green fluorescent nanoparticles (NPs) are used to evaluate the point spatial resolution function (PSF) of nanosheet wrapping mount [21]. From the cross-sectional images of NPs at different depths, we find that an aberration is induced by RI mismatch even though the gel has been treated by LUCID. The full width at half maximum intensity (FWHM) along the intensity profile is measured. While the FWHM_*lateral*_ seems constant at about 250 nm over the whole *z* range, the signal intensity is lost with focusing depth. The FWHM_*axial*_ degrades from about 890 nm at the surface of the nanosheet (*z* = 0), to 1060 nm at *z* = 100 μm, and 1620 nm at *z* = 200 μm (Fig 5B). Our results support the fact that the RI mismatch effect becomes pronounced with increasing the focusing depth [22]. The nanosheet wrapping mount appears to be a possible method to look deeper into a tissue, but some new challenges arise from the requirement to correct optical aberrations.

The mismatched RI can be optimized by adjusting the mounting media of the specimen. We adjust the RI of the gel from 1.496 to 1.515 by further modification with another clearing agent RapiClear, to make the specimen have the same RI as the immersion oil [23]. In this case, the signal intensity is much improved, and the lateral and axial FWHMs stay constant over the whole *z* range at 220 nm and 770 nm, respectively (Fig 5C and 5D). The RapiClear treated gel is observed with the conventional coverslip setup as well. Spatial resolution analysis verifies that with an ideal optical condition without RI mismatch, coverslip setup gives almost the same lateral and axial FWHMs (S3 Fig). And thus, we conclude that by adjusting the refractive index appropriately, nanosheet wrapping mount can provide an equivalently high resolution as that of coverslip setup, even approaching the equipment limit of the resolution of a confocal fluorescence microscopy with a specific high magnification objective lens.

**Fig 6:**
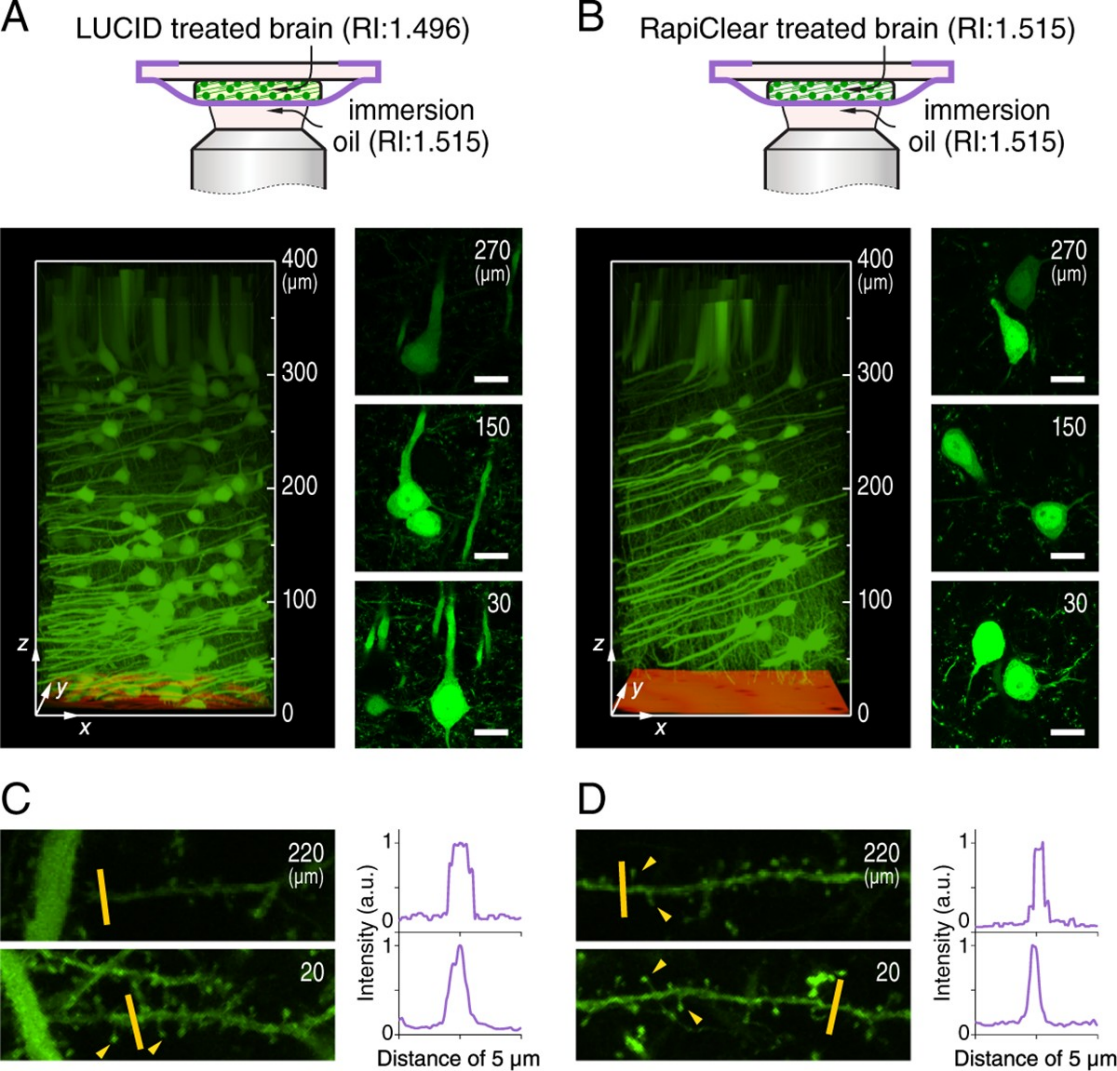
Nanosheet wrapping imaging on a brain slice. (A, B) With nanosheet wrapping mount, a 1 mm thick thy1-EYFP-H mouse brain slice is observed from the surface of nanosheet to a depth of 400 μm, and a 1 μm thick polystyrene film loaded with Nile red is applied as a height reference at *z* = 0. Imaging tests with brain treated with LUCID, and further modified with RapiClear are conducted (*n* = 5 independent tests; and representative images are shown). The corresponding schematics of optics conditions are shown above. Imaging area in *xy* plane is 212×212 μm^2^, and depth is labelled at the side. Images of individual axon fibers at representative depths are extracted and shown at right. Scale bar, 15 μm; and depth is labelled at upper right. (C, D) Magnified views of dendrites are trimmed from projection images (maximum intensity projection) of Fig 6A, Fig 6B at both superficial (depth: 20±10 μm) and deep (depth: 220±10 μm) regions within volume of a 30×10×3 μm^3^ cube. The normalized fluorescence intensity along the yellow lines with a distance of 5 μm is measured to demonstrate the edge of dendrites, and yellow arrows indicate some representative dendritic spines with fine structures.

The performance of nanosheet wrapping mount is further demonstrated with a tissue specimen. A LUCID cleared brain slice from thy1-EYFP-H transgenic mice expressing the enhanced yellow fluorescent protein is used [24], and the neuron cells at the internal pyramidal layer of the cerebral cortex are observed (S4 Fig). Because of the RI mismatch, the brightness and resolution of the image deteriorates as the focus gets deeper (Fig 6A and 6C). After the RI of the specimen is adjusted to 1.515 by RapiClear, fine structures, including individual axon fibers and dendrites, are sharply visualized at multiple depths along the whole 300 μm *z* range (Fig 6B and 6D).

It worth noting that in a practical imaging task, to adjust the RI of different tissues to 1.515 is always cumbersome and can even be impossible. Alternatively, the RI of the immersion liquid can be adjusted to that of the specimen to produce a space-invariant PSF. Here, we choose 2,2’-thiodiethanol (TDE) as an immersion liquid for the objective lens, which can provide a wide RI range from 1.333 to 1.515 by mixing with water [25, 26]. The RI of 87 vol% TDE is 1.496, the same as that of LUCID treated gel and brain slice (S5 Fig). While the signal intensity decreases to some extent, the FWHM_*lateral*_ and FWHM_*axial*_ over the whole *z* range are about 260 nm and 1460 nm, respectively (S6A Fig). The difference in RI from that of the designed objective immersion oil produces aberration, which has been reported previously [27]. After a 3D deconvolution processing with the measured PSF using a commercial or an open-source software [28, 29], the imaging quality is much improved (S6B and S6C Fig). One can see the shape of dendrites and even the dendritic spines at both superficial and deep regions are easily recognized, achieving a high resolution comparable to that of Fig 6B and 6D.

Another remark comes from the correction collar of objective lens. Correction collar is most equipped on high magnification objectives (always for dry and water-immersion objectives) to correct the spherical aberration induced by variation of coverslip thickness and RI mismatch. It is known that even variation of a few micrometers in coverslip thickness can impact in the image quality, especially for high NA objectives [30]. In fact, as the typical coverslip has a RI of 1.515 similar to that of immersion oil, the correction collar on oil-immersion objectives is likely more relevant to address the problem of RI mismatch from specimen aspect. Our results in S3 Fig also show that if a homogenous RI is adjusted, the spatial resolution keeps constant even lack of a coverslip in the optical path. For objectives with other immersion liquids, correction collar plays a more important role in correcting the coverslip thickness variation, typically between 0.13 and 0.22 mm. We compare the spatial resolution between conventional coverslip setup and nanosheet wrapping mount by imaging a specimen with RI of 1.333 using a 60× water-immersion objective lens, which shows that the imaging quality of nanosheet wrapping much deteriorates compared to that of coverslip setup (S7 Fig). It is reasonable to expect such a result as the water-immersion objective lens is designed based on the assumption that a 170 μm thick coverslip is in the optical path. Taken together, the present nanosheet wrapping method is extremely feasible for oil-immersion objectives without too much concern about correction collar. For other immersions, one has to choose no coverslip (NCG) objectives, water dipping objectives, or setting the correction collar to 0 to account for the lack of a coverslip, if possible.

The space-variant aberration can be restored by some deblurring algorithms [31], corrected by adaptive optics techniques [32, 33], or reduced by use of objectives with a motorized correction collar. However, as we indicated above, to adjust RIs homogeneously is a straightforward and effective method to obtain deep tissue imaging with nanosheet wrapping a cleared tissue specimen. In addition, in a routine imaging task, the mounting media gradually evaporates from the surface of tissue, leading to a subtle gradient in its RI and impairing the imaging quality [34]. With nanosheet wrapping, owing to the hydrophobicity of CYTOP, we find about 60% of water inside the tissue can be protected from evaporation even after 24 h, which suggests that the nanosheet wrapped tissue can be kept fresh for a longer time and so avoid RI inhomogeneity (S8 Fig).

## Conclusions

In summary, a coverslip-free imaging method is developed by use of polymer nanosheet wrapping to increase the maximum focusing depth into a cleared tissue specimen. The observable depth breaks the usual limited working distance of an objective lens, and high resolution imaging can be achieved if a homogenous RI between immersion liquid and mounting media is effected by suitable adjustment. As we all know, various super-resolution microscopy techniques have been developed over the past decade, such as stimulated emission depletion microscopy (STED), saturated structured illumination microscopy (SSIM), spectral precision distance microscopy (SPDM), photo activated localization microscopy (PALM), stochastic optical reconstruction microscopy (STORM), etc. [35–39] In a seminal review, Alivisatos et al. discussed a number of recently developed nanotools for use in neuroscience and brain activity mapping, and pointed out that nanoscience and nanotechnology are poised to provide a rich toolkit of novel methods in biological research work [40]. Compared to the rapid development of microscopy, we find the specimen mounting technique leaves much space to be improved, which can be addressed using a film-shaped nanomaterial, i.e., nanosheet. While the method proposed in this study was tested with a confocal fluorescence microscopy only, we anticipate that a combination with the existing super-resolution microscopy techniques will further broaden its applicability.

## Supporting information

S1 Text Protocol for nanosheet wrapping-assisted coverslip-free tissue imaging. (PDF)

S1 Fig Thickness of prepared CYTOP nanosheets. Correlation between the thickness of CYTOP nanosheet and the concentration of coating solution (*n* = 9). Arrow shows the 30 mg mL^–1^ solution used in this study gives a thickness of 130 nm. As the error bars are obscured by the data point for some low concentrations, the original film thickness date shown in mean ± SD are given in a table as well.
(TIF)

S2 Fig Transparence of CYTOP nanosheets. (A) Photo of a thy1-EYFP-H transgenic mouse brain slice (1mm thickness, treated with LUCID) wrapped with a nanosheet. (B) Transmittance over the wavelength range from 300 to 800 nm of a CYTOP nanosheet with a thickness of 130 nm.
(TIF)

S3 Fig Spatial resolutions at different depths of conventional coverslip setup. 200 nm NPs are embedded in agarose gel treated with RapiClear and images are obtained at every 50 μm. Scale bar, 500 nm; and depth is labelled below. The corresponding schematics of optics conditions are shown above. Representative NPs at each depth are arbitrarily chosen (*n* = 3 independent tests; and 10 NPs are analyzed for each test), and FWHMs along the intensity profile in *x*-axis and *z*-axis are measured as shown in histograms (mean ± SEM).
(TIF)

S4 Fig Clearing of thy1-EYFP-H transgenic mouse brain slices. (A) Photos of brain slices (1 mm thick) before clearing, after treated with LUCID, and after further modified with RapiClear (from top to down). (B) Fluorescence image of a whole brain slice with tiled scanning to indicate the location where the neuron cells are observed for deep tissue imaging test in this study, as highlighted in the box. Inset is a magnified view of this location.
(TIF)

S5 Fig Correlation between RI and TDE concentration. Arrows show the concentration of 87 vol% TDE gives a RI of 1.496.The measurement is conducted under 589 nm at 23°C.
(TIF)

S6 Fig Coverslip-free imaging on a LUCID treated brain slice with 87% TDE as immersion liquid. (A) With nanosheet wrapping mount, an agarose gel embedded with 2 μm green fluorescent beads is observed from the upper surface of coverslip to a depth of 400 μm, and a 1 μm thick polystyrene film loaded with Nile red is applied as a height reference at *z* = 0. The corresponding schematic of optics condition is shown above, and yellow dashed line indicates the maximum depth of focal plane. Imaging area in *xy* plane is 212×212 μm^2^; and depth is labelled at the side. Spatial resolutions at different depths of nanosheet wrapping mount are shown at right, where 200 nm NPs are embedded in agarose gel and images are obtained at every 50 μm. Scale bar, 500 nm; and depth is labelled below. Representative NPs at each depth are arbitrarily chosen (*n* = 3 independent tests; and 10 NPs are analyzed for each test), and FWHMs along the intensity profile in *x*-axis and *z*-axis are measured as shown in histograms (mean ± SEM). (B) A thy1-EYFP-H mouse brain slice (1 mm thick) is observed from the surface of nanosheet to a depth of 400 μm, and a 1 μm thick polystyrene film loaded with Nile red is applied as a height reference (*n* = 5 independent tests; and typical images are shown). (C) The raw data of S5B Fig has been processed with 3D deconvolution using the measured PSF (S5A Fig). It worth noting that due to the brightness information has been normalized during a deconvolution process; it is not meaningful to compare the signal intensity of S5C Fig with S5B Fig. And thus, we adjust the brightness of S5C Fig arbitrarily for a better representation of the improved signal-to-noise ratio after deconvolution. The corresponding schematic of optics conditions for S5B Fig and S5C Fig are shown above. Imaging area in *xy* plane is 212×212 μm^2^; and depth is labelled at the side. Images of individual axon fibers at representative depths are extracted and shown at right. Scale bar, 15 μm; and depth is labelled at upper right. Magnified views of dendrites are trimmed from projection images (maximum intensity projection) at both superficial (depth: 20±10 μm) and deep (depth: 220±10 μm) regions within volume of a 30×10×3 μm^3^ cube, as shown below. The normalized fluorescence intensity along the yellow lines with a distance of 5 μm is measured to demonstrate the edge of dendrites, and yellow arrows indicate some representative dendritic spines with fine structures.
(TIF)

S7 Fig Coverslip-free imaging on a model tissue with a water-immersion objective lens. (A, C) With coverslip setup and nanosheet wrapping mount, imaging test with an agarose gel embedded with 2 μm green fluorescent beads is conducted. Images are taken from the upper surface of coverslip or nanosheet to a depth of 500 μm, and a 1 μm thick polystyrene film loaded with Nile red is applied as a height reference at *z* = 0. The corresponding schematics of optics conditions are shown above, and yellow dashed lines indicate the maximum depth of focal plane in each condition. Imaging area in *xy* plane is 212×212 μm^2^, and depth is labelled at the side. (B, D) Spatial resolutions at different depths of coverslip setup and nanosheet wrapping mount. 200 nm NPs are embedded in agarose gel and images are obtained at every 50 μm. Scale bar, 500 nm; and depth is labelled below. Representative NPs at each depth are arbitrarily chosen (*n* = 3 independent tests; and 10 NPs are analyzed for each test), and FWHMs along the intensity profile in *x*-axis and *z*-axis are measured as shown in histograms (mean ± SEM).
(TIF)

S8 Fig Water retention ability of nanosheet wrapping. (A) Correlation between the water retention ratio and the time of test for a CYTOP nanosheet (thickness of 130 nm) wrapped alginate hydrogel (*n* = 3; control group: without nanosheet wrapping). (B) Photos of pristine alginate hydrogel loaded with Blue dextran (diameter: 10 mm; thickness: ~5 mm), nanosheet wrapped hydrogel, and hydrogels after 6, 12, and 24 h test with or without nanosheet wrapping.
(TIF)
