# Supplementary material for: Nanosheet wrapping-assisted coverslip-free imaging for looking deeper into a tissue at high resolution
Source: PLoS One. 2020 Jan 10;15(1):e0227650. doi: 10.1371/journal.pone.0227650 (PMC6953877; doi:10.1371/journal.pone.0227650)
Supplement: S1 Text — (PDF) [file pone.0227650.s001.pdf]

Protocol for nanosheet wrapping-assisted coverslip-free tissue imaging

Materials, reagents, and equipment

Materials		
Fresh silicon substrates (35×35 mm^2) or silicon substrates washed by piranha solution		
Homebuilt circular wire loop (*ca.* 2–3 mm larger than the diameter of coverslip)		
Perforated Petri dish with an opening hole *ca.* 2–3 mm smaller than the diameter of coverslip		
Glassware, coverslip, petri dish, tweezers, etc.		
Reagents		
Chemicals name	*Solvent*	*Concentration*
Poly(vinyl alcohol) (PVA)	Water	10 mg mL^{-1}
CYTOP (CTX-809SP)	Perfluorotributylamine	30 mg mL^{-1}
Tissue clearing agents	--	--
Equipment		
Spin-coater		
Inverted confocal microscopy with a high NA oil-immersion objective lens		

Procedure

1. Drop the PVA solution on silicon substrate and spin-coat at 4000 rpm for 60 s;
2. Drop the CYTOP solution on PVA coated substrate and spin-coat at 4000 rpm for 60 s;
3. Immerse the coated substrate into water to obtain CYTOP nanosheet floating on water surface;
4. Scoop the nanosheet out of water using a circular wire loop and dry in the air;
5. Place the cleared tissue specimen in the center of a coverslip;
6. Wrap the tissue using the wire loop supported nanosheet from above, until the fracture of nanosheet occurs as it contacts the edge of coverslip;
7. Reverse the wrapped specimen and gently place it into the center of a perforated Petri dish;
8. Fix the Petri dish on the stage of microscopy and observe with a high NA oil-immersion lens.

Notes

1. Tissue specimen should be cleared according to the protocol of a specific clearing agent, such as Sca*l*eS, 3DISCO, CLARITY, SeeDB, *Clear*^{T}, CUBIC, LUMOS, LUCID, RapiClear, etc. before wrapping;
2. If needed, an immersion liquid should be chosen to have the same refractive index with the cleared tissue specimen, such as TDE immersion with adjustable refractive index.
