# Supplementary figures and images for: Nanosheet wrapping-assisted coverslip-free imaging for looking deeper into a tissue at high resolution
Source: PLoS One. 2020 Jan 10;15(1):e0227650. doi: 10.1371/journal.pone.0227650 (PMC6953877; doi:10.1371/journal.pone.0227650)

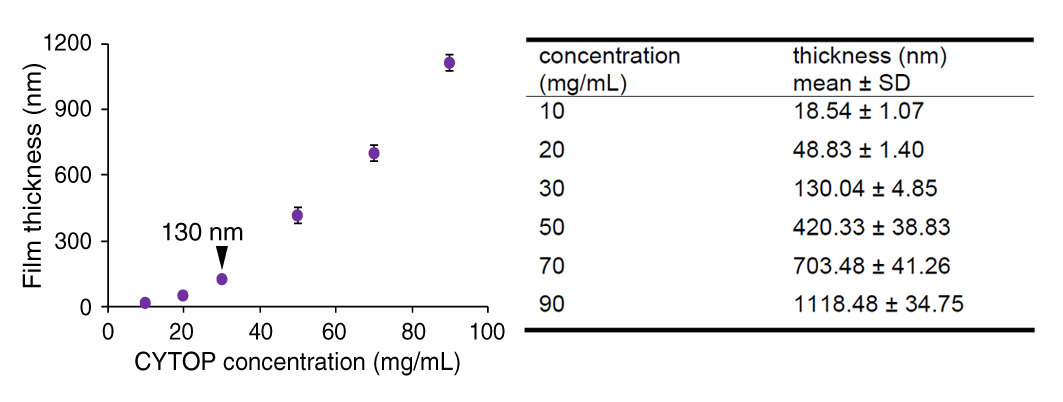

Supplement: S1 Fig — Correlation between the thickness of CYTOP nanosheet and the concentration of coating solution (n = 9). Arrow shows the 30 mg mL–1 solution used in this study gives a thickness of 130 nm. As the error bars are obscured by the data point for some low concentrations, the original film thickness date shown in mean ± SD are given in a table as well. (TIF) [file pone.0227650.s002.tif]

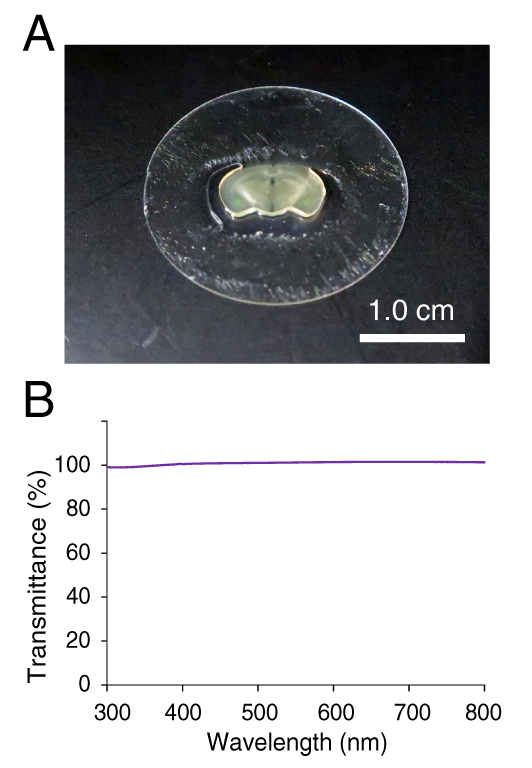

Supplement: S2 Fig — (A) Photo of a thy1-EYFP-H transgenic mouse brain slice (1mm thickness, treated with LUCID) wrapped with a nanosheet. (B) Transmittance over the wavelength range from 300 to 800 nm of a CYTOP nanosheet with a thickness of 130 nm. (TIF) [file pone.0227650.s003.tif]

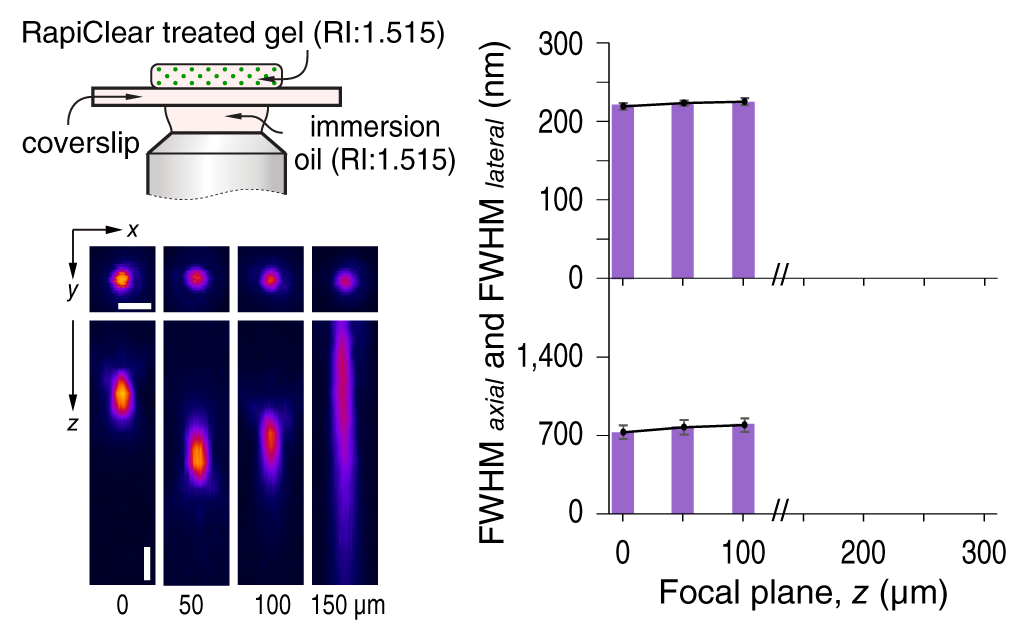

Supplement: S3 Fig — 200 nm NPs are embedded in agarose gel treated with RapiClear and images are obtained at every 50 μm. Scale bar, 500 nm; and depth is labelled below. The corresponding schematics of optics conditions are shown above. Representative NPs at each depth are arbitrarily chosen (n = 3 independent tests; and 10 NPs are analyzed for each test), and FWHMs along the intensity profile in x-axis and z-axis are measured as shown in histograms (mean ± SEM). (TIF) [file pone.0227650.s004.tif]

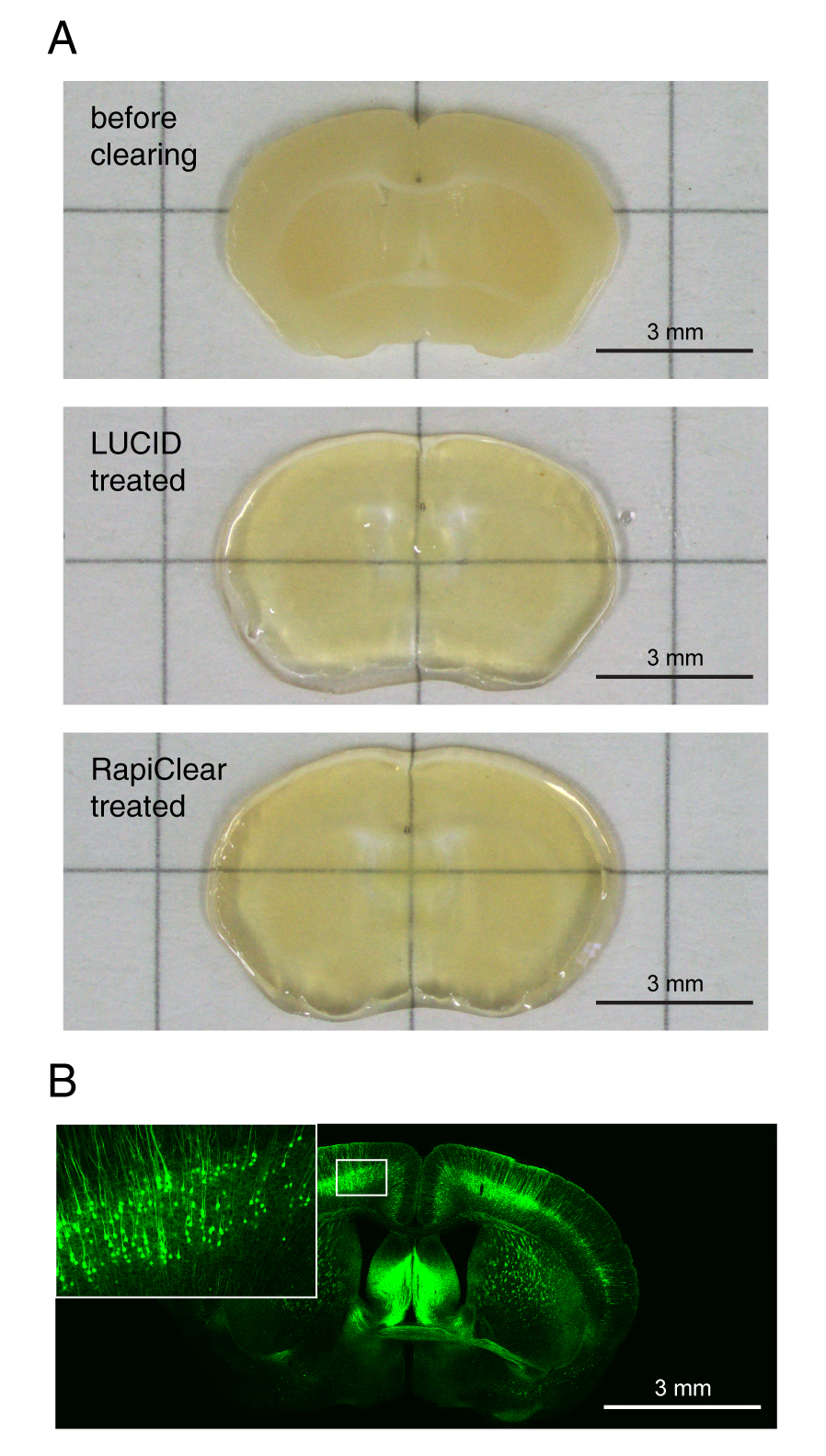

Supplement: S4 Fig — (A) Photos of brain slices (1 mm thick) before clearing, after treated with LUCID, and after further modified with RapiClear (from top to down). (B) Fluorescence image of a whole brain slice with tiled scanning to indicate the location where the neuron cells are observed for deep tissue imaging test in this study, as highlighted in the box. Inset is a magnified view of this location. (TIF) [file pone.0227650.s005.tif]

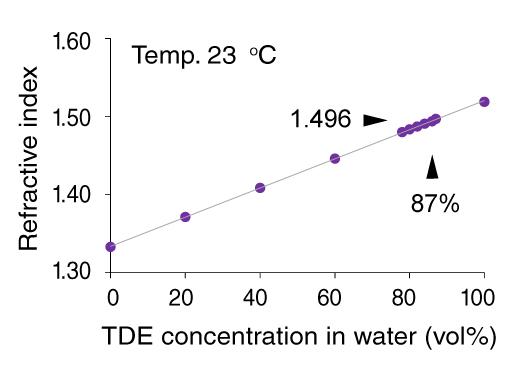

Supplement: S5 Fig — Arrows show the concentration of 87 vol% TDE gives a RI of 1.496.The measurement is conducted under 589 nm at 23°C. (TIF) [file pone.0227650.s006.tif]

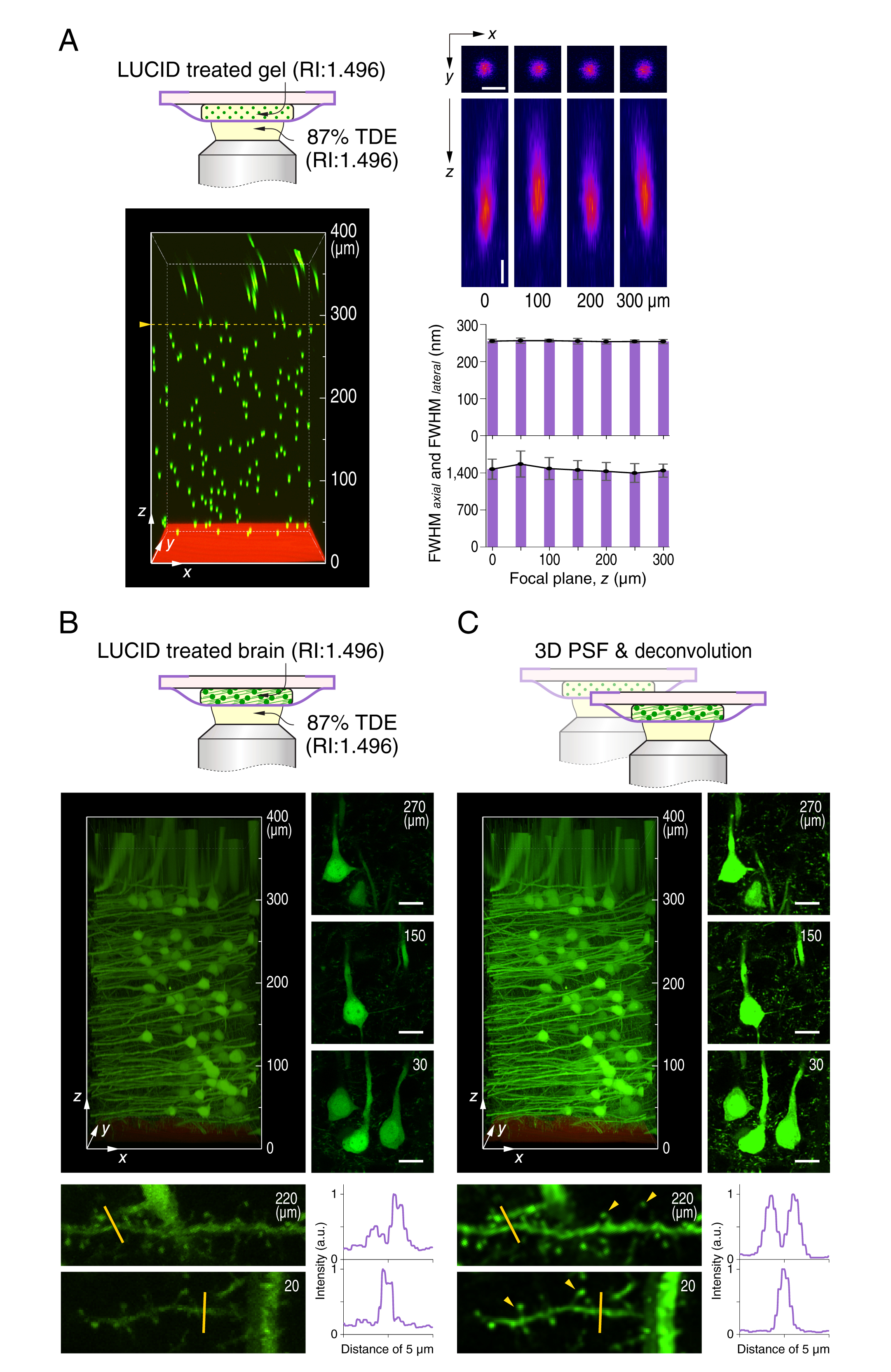

Supplement: S6 Fig — (A) With nanosheet wrapping mount, an agarose gel embedded with 2 μm green fluorescent beads is observed from the upper surface of coverslip to a depth of 400 μm, and a 1 μm thick polystyrene film loaded with Nile red is applied as a height reference at z = 0. The corresponding schematic of optics condition is shown above, and yellow dashed line indicates the maximum depth of focal plane. Imaging area in xy plane is 212×212 μm2; and depth is labelled at the side. Spatial resolutions at different depths of nanosheet wrapping mount are shown at right, where 200 nm NPs are embedded in agarose gel and images are obtained at every 50 μm. Scale bar, 500 nm; and depth is labelled below. Representative NPs at each depth are arbitrarily chosen (n = 3 independent tests; and 10 NPs are analyzed for each test), and FWHMs along the intensity profile in x-axis and z-axis are measured as shown in histograms (mean ± SEM). (B) A thy1-EYFP-H mouse brain slice (1 mm thick) is observed from the surface of nanosheet to a depth of 400 μm, and a 1 μm thick polystyrene film loaded with Nile red is applied as a height reference (n = 5 independent tests; and typical images are shown). (C) The raw data of S5B Fig has been processed with 3D deconvolution using the measured PSF (S5A Fig). It worth noting that due to the brightness information has been normalized during a deconvolution process; it is not meaningful to compare the signal intensity of S5C Fig with S5B Fig. And thus, we adjust the brightness of S5C Fig arbitrarily for a better representation of the improved signal-to-noise ratio after deconvolution. The corresponding schematic of optics conditions for S5B Fig and S5C Fig are shown above. Imaging area in xy plane is 212×212 μm2; and depth is labelled at the side. Images of individual axon fibers at representative depths are extracted and shown at right. Scale bar, 15 μm; and depth is labelled at upper right. Magnified views of dendrites are trimmed from projection images (maximum in [file pone.0227650.s007.tif]

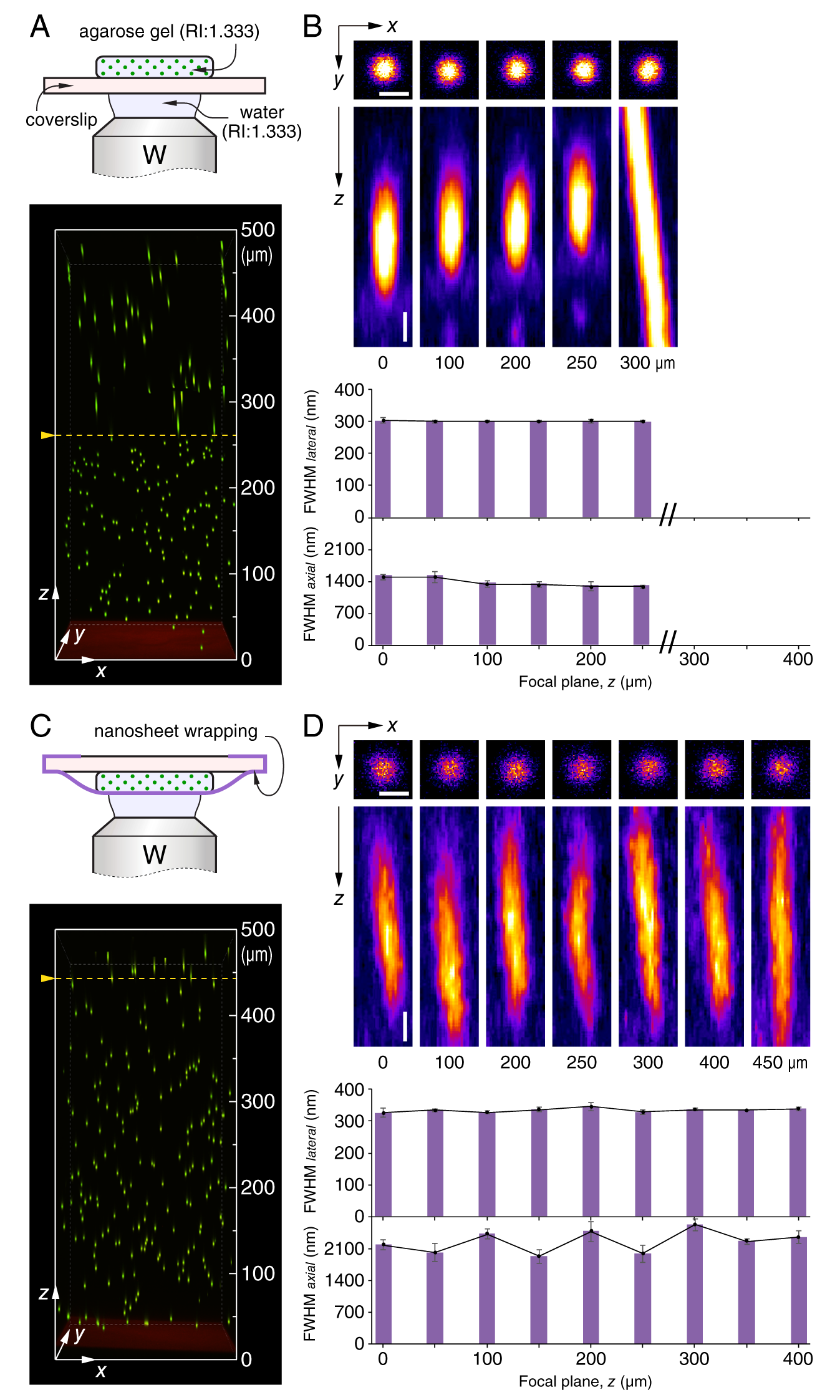

Supplement: S7 Fig — (A, C) With coverslip setup and nanosheet wrapping mount, imaging test with an agarose gel embedded with 2 μm green fluorescent beads is conducted. Images are taken from the upper surface of coverslip or nanosheet to a depth of 500 μm, and a 1 μm thick polystyrene film loaded with Nile red is applied as a height reference at z = 0. The corresponding schematics of optics conditions are shown above, and yellow dashed lines indicate the maximum depth of focal plane in each condition. Imaging area in xy plane is 212×212 μm2, and depth is labelled at the side. (B, D) Spatial resolutions at different depths of coverslip setup and nanosheet wrapping mount. 200 nm NPs are embedded in agarose gel and images are obtained at every 50 μm. Scale bar, 500 nm; and depth is labelled below. Representative NPs at each depth are arbitrarily chosen (n = 3 independent tests; and 10 NPs are analyzed for each test), and FWHMs along the intensity profile in x-axis and z-axis are measured as shown in histograms (mean ± SEM). (TIF) [file pone.0227650.s008.tif]

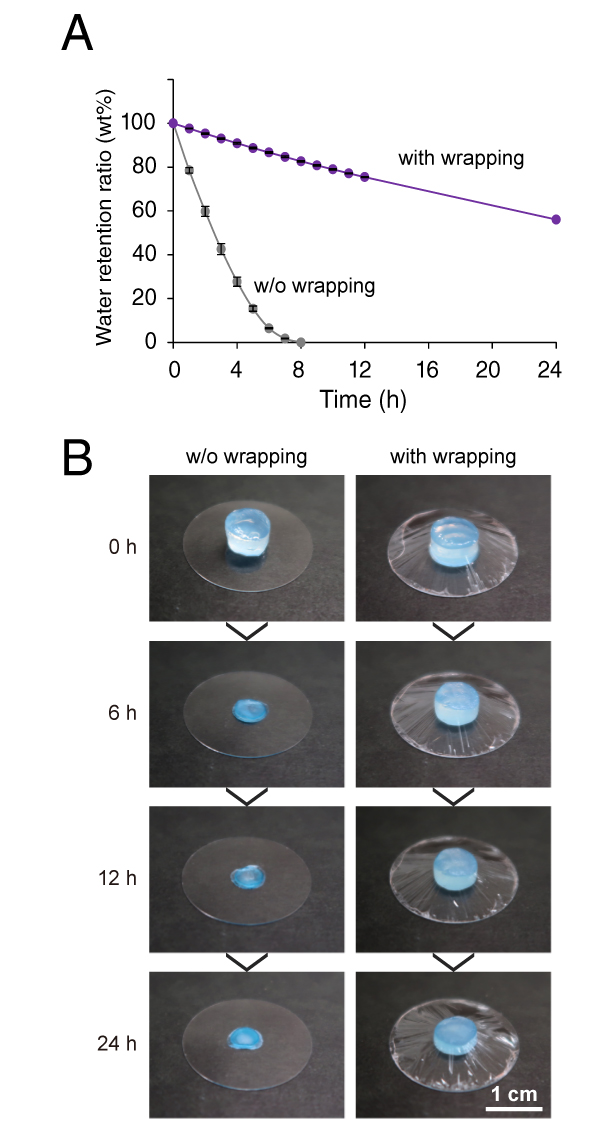

Supplement: S8 Fig — (A) Correlation between the water retention ratio and the time of test for a CYTOP nanosheet (thickness of 130 nm) wrapped alginate hydrogel (n = 3; control group: without nanosheet wrapping). (B) Photos of pristine alginate hydrogel loaded with Blue dextran (diameter: 10 mm; thickness: ~5 mm), nanosheet wrapped hydrogel, and hydrogels after 6, 12, and 24 h test with or without nanosheet wrapping. (TIF) [file pone.0227650.s009.tif]
